# An Unusual Presentation of Influenza-Induced Myositis

**DOI:** 10.7759/cureus.13196

**Published:** 2021-02-07

**Authors:** Mohamed M Elagami, Moutaz Ghrewati, Ibrahim Khaddash, Gabriel Melki

**Affiliations:** 1 Internal Medicine, St. Joseph's Regional Medical Center, Paterson, USA

**Keywords:** influenza, myositis, inflammation, enzymes, serologic

## Abstract

Post-influenza myositis is considered a distinct clinical entity and is associated with muscle pain and elevated muscle enzymes during convalescence. Although the exact mechanism of muscle injury in acute viral myositis is unknown, there are possible mechanisms proposed in the literature. The progression of viral myositis to rhabdomyolysis, although uncommon, can be life-threatening and has been reported with many viruses, most commonly influenza. At our institution, a case of severe influenza-induced myositis prompted us to conduct a literature search focusing on the incidence, pathophysiology, typical presentation, and proper diagnosis of this rare condition.

## Introduction

Myositis is a disease of muscle inflammation that typically results in muscular injury. Etiologies of myositis include medication-induced myopathies, endocrinopathies, electrolyte disorders, infections, autoimmune, neuromuscular, and rheumatologic disorders. Influenza is the most common viral etiology of myositis and rhabdomyolysis, followed by HIV and enteroviral infections. Mild to moderate myalgia is a common feature of viral illness and rarely progresses to myositis or rhabdomyolysis. This patient-centered review shows the importance of high clinical suspicion for influenza-induced myositis when the presenting illness is preceded by an upper respiratory infection alongside myalgia, muscular tenderness on examination, and evidence of muscle injury on laboratory workup.

## Case presentation

A 38-year-old Jordanian male with a past medical history of absence seizure presented with a three-day history of flu-like symptoms including fever, severe generalized weakness, headache, diffuse myalgia of the neck, back and proximal muscles of the limbs, nasal congestion, cough productive of white sputum, and poor appetite. His only home medication was carbamazepine 200mg every 12 hours. Physical examination did not reveal any abnormalities apart from a temperature of 40 degrees Celsius. Initial blood work was unremarkable, except for elevation of erythrocyte sedimentation rate (ESR) (30) [reference range 0-10 mm/hour], and C-reactive protein (CRP) (140) [reference range 0-10 mg/L]. Urinalysis (UA) was significant for large blood with no red blood cells that could be identified. This initial investigation raised suspicion for the presence of muscle breakdown, for which additional laboratory work was ordered (Table 1).

**Table 1:** Enzymes

Name of test	Reading	Reference range
Creatinine Phosphokinase (CPK)	58,824 units/L	30 -223
Aspartate aminotransferase (AST)	374 unit/L	13 – 39
Alanine aminotransferase (ALT)	49 unit/L	17 – 52
Lactate Dehydrogenase (LDH)	634 unit/L	140 – 271
Aldolase	94 unit/L	3.3 – 10.3

Based on the results in Table 1, the diagnosis of myositis was confirmed, and supportive care was initiated with intravenous (IV) fluid hydration using 0.9% normal saline as well as analgesics. Meanwhile, a secondary workup was obtained to identify the etiology. A urine toxicology study, blood cultures, a hepatitis profile and a rapid influenza antigen serology test were all obtained. The results were unremarkable except for the presence of influenza A antigen. The patient was then treated with oseltamivir phosphate (Tamiflu) in addition to the supportive measures. Due to the patient’s history of seizures, an electroencephalogram was conducted and was negative for any seizure activity.

Thus, this patient had a very rare complication of influenza that led to myositis, which improved with Tamiflu and supportive care. The patient was hospitalized for a total of seven days, successfully recovered, and was seen by our medical team in the outpatient clinic one month following discharge from the hospital.

## Discussion

Myositis is a disease of muscle inflammation which typically manifests as myalgia that is often symmetrical and involves the proximal muscles [1]. Patients often complain of muscle weakness on presentation that may be generalized or localized. The differential diagnosis for weakness is broad and may include toxic myopathies (alcohol, cocaine), endocrine disorders (thyroid disease, Cushing syndrome, Addison’s disease), electrolytes disorders (hypokalemia, hypocalcemia, hypercalcemia, hypomagnesemia), autoimmune, neuromuscular and rheumatologic disorders, medications (statins, bisphosphonates, ciprofloxacin), and infections (viral, bacterial, parasitic) [1].

Post-influenza myositis is considered a distinct clinical entity and is associated with muscle pain and elevated muscle enzymes during convalescence [2]. Mild to moderate myalgia is a common symptom in acute viral infection. Patients commonly complain of back and leg pain, and examination may reveal muscle tenderness and edema [3]. Symptoms are often self-limited. However, in cases where myalgia progresses to myositis and rhabdomyolysis, muscle involvement is diffuse, and the weakness reported by patients is more severe [4].

The exact mechanism of muscle injury in acute viral myositis is unknown. However, there are possible mechanisms as proposed in the literature. The first possibility is that there is direct invasion of muscle tissue by the virus and thus direct toxic effect on muscle cells [2]. Alternatively, there may be an immunologic process triggered by the viral infection that ultimately causes muscle damage without directly infecting the muscle [4]. Muscle injury as a result causes liberation of muscle enzymes including creatinine phosphokinase (CPK), aldolase, and other intracellular enzymes such as aspartate aminotransferase (AST), alanine aminotransferase (ALT), and lactate dehydrogenase (LDH) [3]. Progression of viral myositis to rhabdomyolysis, although uncommon, can be life-threatening and has been reported with the following viruses: influenza A and B, enteroviruses, human immunodeficiency virus (HIV), human T-cell leukemia-lymphoma virus (HTLV) type 1, hepatitis viruses B and C [4].

The diagnosis of viral myositis and the identification of its etiology starts with a detailed history and careful physical examination. A complete blood count, urinalysis, and measures of renal and liver function are also appropriate for most patients with significant myalgia. Certain clinical situations will require blood cultures, serologic testing for infectious agents, erythrocyte sedimentation rate (ESR), C-reactive protein (CRP), or autoantibody levels (antinuclear antibody [ANA], rheumatoid factor [RF], antineutrophil cytoplasmic antibodies [ANCA], anti-cyclic citrullinated peptide [anti-CCP]) [5].

Influenza is the most common viral etiology of myositis and rhabdomyolysis, followed by HIV and enteroviral infections [6]. Infections with seasonal influenza A and B are common in the winter months in temperate climates. However, in tropical and subtropical climates it occurs year-round and peaks during cooler or rainy-season months [7]. It is important, therefore, to rule out an inflammatory muscle process such as myositis or rhabdomyolysis in patients who present with acute viral infection such as influenza, particularly when symptoms include moderate to severe myalgia.

To diagnose viral-induced myositis, clinicians should look for a history consistent with flu symptoms (fever, cough, rhinorrhea, headache, weakness) along with myalgia, physical examination that reveals muscle tenderness and lab work aimed at ruling out muscle inflammation or injury such as urinalysis (myoglobinuria), AST, ALT, LDH, CPK, and aldolase levels. Additional testing of clinical specimens (nasopharyngeal swab, serology, stool specimen, and/or cerebrospinal fluid) may) have a greater likelihood of establishing a definitive viral etiology if done early in the course of the infection. Muscle biopsy is not indicated in the setting of suspected viral myositis; however, it may be used to exclude other causes of inflammatory myopathies if necessary [3].

## Conclusions

The diagnosis of influenza-induced myositis can easily be overlooked if clinicians focus on symptomatic treatment of this viral infection without consideration of its complications with respect to muscle inflammation and/or muscle injury. Clinicians should have high clinical suspicion when they come across a history of present illness that is consistent with precedent viral infection along with myalgia, a physical examination that reveals muscle tenderness and lab work that shows evidence of muscle injury in a patient with influenza virus infection. A focused history and physical examination as well as ordering pertinent laboratory work (UA, kidney and liver profile, LDH, CPK, and aldolase levels) should help with prompt diagnosis and treatment of this rare condition.
